# Foreword by the Secretariat of the WHO Framework Convention on Tobacco Control

**DOI:** 10.1136/tobaccocontrol-2018-054882

**Published:** 2019-06-18

**Authors:** Vera Luiza da Costa e Silva

**Affiliations:** Convention Secretariat, WHO FCTC & Protocol to eliminate Illicit Trade in Tobacco Products, Organisation mondiale de la Sante, Geneve, Switzerland

**Keywords:** global health, nicotine, public policy, secondhand smoke, tobacco industry

The WHO Framework Convention on Tobacco Control (WHO FCTC) is one of the biggest public health achievements of the 21st century. It is the world’s first public health treaty negotiated under the WHO constitutional framework and is today legally binding in 181 Parties (180 countries and the European Union). The Convention aims to protect present and future generations from the devastating health, economic, social and environmental impact of tobacco and requires implementation of a comprehensive set of supply and demand reduction measures to this effect. It is a strong public health instrument to the effect that it entails the involvement of a number of different sectors for its implementation and a combination of cross-cutting provisions, such as Article 5.3 on protection of public health policies from tobacco industry interference and Article 19 on liability as a public health measure.

The Conference of the Parties is the governing body of the WHO FCTC, the world’s only intergovernmental body fully responsible for shaping global directions in tobacco control. Through its decisions, the Conference of the Parties guides the Parties to the Convention in their implementation work and facilitates engagement with other stakeholders at regional and global levels to mobilise support for the implementation of the Convention. Its decisions and the guidelines on various WHO FCTC articles that it adopted provide guidance to Parties on how to implement strong policies and legislation in line with the requirements of the Convention, thus addressing an epidemic that claims globally 7 million deaths each year, due to either tobacco use or exposure to secondhand smoke.

The WHO FCTC had been in force for almost 10 years, when the Conference of the Parties, in late 2014 at its sixth session held in Moscow, Russian Federation, decided to assess its impact.

The WHO FCTC impact assessment was carried out by an expert group of seven members with support from the Convention Secretariat, through desk research and country missions organised to 12 Parties (Bangladesh, Brazil, Islamic Republic of Iran, Kenya, Madagascar, Pakistan, Philippines, Republic of Korea, Sri Lanka, Turkey, UK of Great Britain and Northern Ireland and Uruguay) to the Convention. The countries visited were actually selected by the expert group itself, taking into account a balanced representation of different parts of the world. The assessment provided an opportunity to reveal what difference the WHO FCTC has made in shaping the countries’ tobacco control policies and to measure its effectiveness as a tool to reduce tobacco consumption and prevalence. All in all, the final expert report provided an overview on how the Convention has promoted the inclusion of tobacco control in the political agenda, supporting at the same time a multisectoral approach across different governmental sectors.

An impact assessment constitutes in itself an invaluable analytical instrument. It can be used as part of the regular monitoring of WHO FCTC implementation, thus incorporating the study of social and economic impact of tobacco use in addition to the standard surveillance of tobacco use and exposure to tobacco smoke. Therefore, it represents one of the approaches for implementing Article 20 of the Convention (*Research, surveillance and exchange of information*). In principle, it is advisable to conduct baseline data collection at the country level before the adoption of tobacco control policies, followed by impact assessment studies after the implementation of policies.

This supplement disseminates the findings of the impact assessment and provides the academic community with an example of how a treaty body can promote research and monitoring initiatives under its framework. It also indicates ways in which global initiatives that include the implementation of the WHO FCTC as targets can consider monitoring strategies to assess progress in reaching their goals.

The 2030 Agenda for Sustainable Development Goal (SDG) recognises the importance of tobacco control as key to sustainable development and incorporates implementation of the WHO FCTC as one of the SDGs (target 3.a).

Additionally, global efforts to strengthen control and prevention of non-communicable diseases (NCDs) could serve as a vehicle to reinforce Parties’ commitments to full implementation of WHO FCTC. As tobacco use is responsible for one in six deaths from NCDs, it is critical to comprehensively integrate the Convention into global initiatives heading towards the achievement of the SDGs, particularly target 3.4, as well as the control of NCDs. The 2018 Political Declaration on NCDs adopted at the High Level Meeting on NCDs on September 2018, reflects countries’ commitment to accelerate the implementation of the WHO FCTC and the ratification by those that are not yet Parties, and at the same time prevent tobacco industry interference with tobacco control initiatives in accordance with Article 5.3 of the Convention.

Implementation of the WHO FCTC could also serve as a critical ingredient in the control of some communicable diseases where tobacco is an additional risk factor and a cause of sometimes severe comorbidities. Evidence from recent research demonstrates strong links between tobacco use and the increased risks of tuberculosis and HIV/AIDS infection.

The impact assessment exercise has also helped to shape a number of international developments under the WHO FCTC umbrella. The eighth session of the Conference of the Parties has recently adopted the ‘Global Strategy to Accelerate Tobacco Control: Advancing Sustainable Development through Implementation of the WHO FCTC 2019–2025’. The Global Strategy will function as a guide for national priority setting and for the development of programmes and plans for tobacco control. In this context, the impact assessment of previously adopted policies will serve as advocacy tools in strengthening tobacco control policies and full implementation of the Convention.

The impact assessment was a complex undertaking mandated by the Conference of the Parties. The Convention Secretariat would like to express its gratitude to the Expert Group members who made this exercise possible and to the Parties that voluntarily agreed to host missions. We also recognise the contributions of the government WHO FCTC focal points to this global exercise. It is due to their engagement, invaluable contribution and support that this initiative could take place and benefit from their insight.

The WHO FCTC Impact Assessment Group, whose collaboration made this special supplement possible, consisted of the independent impact assessment expert group, the Convention Secretariat, the representatives of Parties that hosted impact assessment missions, consultants who provided support to the expert group during the impact assessment missions and the International Tobacco Control (ITC) Project. They contributed to the managing of the impact assessment exercise and to the elaboration of the scientific papers and policy briefs that were developed during this analysis. The contributions of all listed below are therefore warmly acknowledged:

- Members of the independent WHO FCTC Impact Assessment Expert Group: Professor Mike Daube (Australia), Professor Geoffrey T Fong (Canada), Dr Sudhir Gupta (India), Mr Thomas F McInerney (Italy), Professor Pekka Puska (Finland), Professor Corne van Walbeek (South Africa) and Dr Ghazi Zaatari (Lebanon).
- National focal points of WHO FCTC Parties for the impact assessment mission: Khairul Alam (Bangladesh), Felipe Mendes (Brazil), Behzad Valizadeh (Islamic Republic of Iran), Dorcas Jepsongol Kiptui (Kenya), Aurélien Ravelojoeliandriambeloaritafika (Madagascar), Muhammad Javed (Pakistan), Elizabeth Caluag (Philippines), Youngjin Jang (Republic of Korea), Palitha Abeykoon (Sri Lanka), Peyman Altan (Turkey), Matthew Birkenshaw (UK of Great Britain and Northern Ireland) and Enrique Soto (Uruguay).
- Secretariat of the WHO FCTC & Protocol to Eliminate Illicit Trade in Tobacco Products: Dr Vera Luiza da Costa e Silva (Head of the Convention Secretariat), Dr Tibor Szilagyi (Team Leader, Reporting and Knowledge Management), Lina Sovani, Patrick Musavuli, Leticia Martinez Lopez and Dominique Nguyen (Technical Officers).
- Consultants who provided support to the expert group during the impact assessment missions: Paula Beltran, Daniel Ferrante, Trinette Lee, and Patrick Musavuli.
- ITC Project support team: Dr Janet Chung-Hall, Lorraine Craig, Michelle Bishop, Dana Komer and Dr Genevieve Sansone.
- Authors and/or peer reviewers of the impact assessment background documents: Suzanne Y Zhou, Jonathan Liberman, Evita Ricafort, Shannon Gravely, Natalie Sansone, Samantha Filby, Asma Bazzi and Stella Bialous  

The elaboration of this supplement was coordinated by the ITC Project at the University of Waterloo in Canada (Professor Geoffrey T Fong and Lorraine Craig).

The current supplement comprises nine papers, covering the analysis of the impact of the WHO FCTC in general and in relation to various articles to the Convention as well as in relation to horizontal issues that cut across various articles and measures. The papers in this supplement take into account practices that have the highest impact in implementation of the Convention and highlight challenges, needs and gaps and make recommendations for future actions.

1. Impact Assessment of WHO FCTC: introduction, general findings and discussion.
2. Impact of the WHO FCTC over the first decade: a global evidence review prepared for the Impact Assessment Expert Group.
3. Impact Assessment of the WHO FCTC over its first decade: methodology of the expert group.
4. The impact of the WHO FCTC in defending legal challenges to tobacco control measures.
5. Analysis of Article 6 (tax and price measures to reduce the demand for tobacco products) of the WHO FCTC.
6. Impact of the WHO FCTC on non-cigarette tobacco products.
7. WHO FCTC and global governance: effects and implications for future global public health instruments.
8. Impact of the implementation of the WHO FCTC on the tobacco industry’s behaviour
9. The impact of the WHO FCTC: Perspectives from stakeholders in 12 countries

We hope that this is a useful account-taking of the progress and impact of the WHO FCTC in its first 10 years of operation.

